# Predictive factors of high societal costs among chronic low back pain patients

**DOI:** 10.1002/ejp.1488

**Published:** 2019-10-10

**Authors:** Elizabeth N. Mutubuki, Mariette A. Luitjens, Esther T. Maas, Frank J. P. M. Huygen, Raymond W. J. G. Ostelo, Maurits W. van Tulder, Johanna M. van Dongen

**Affiliations:** ^1^ Department of Health Sciences Faculty of Science Vrije University Amsterdam Amsterdam Movement Sciences Research Institute Amsterdam The Netherlands; ^2^ Child and Adolescent Health Service Perth Australia; ^3^ School of Population and Public Health University of British Columbia Vancouver Canada; ^4^ Department of Anesthesiology Centre of Pain Medicine Erasmus Medical Center Rotterdam The Netherlands; ^5^ Department of Epidemiology and Biostatistics VU University Medical Center Amsterdam Movement Sciences Research Institute Amsterdam The Netherlands; ^6^ Department of Physiotherapy & Occupational Therapy Aarhus University Hospital Aarhus Denmark

## Abstract

**Background:**

Societal costs of low back pain (LBP) are high, yet few studies have been performed to identify the predictive factors of high societal costs among chronic LBP patients. This study aimed to determine which factors predict high societal costs in patients with chronic LBP.

**Methods:**

Data of 6,316 chronic LBP patients were used. In the main analysis, high societal costs were defined as patients in the top 10% of cost outcomes. Sensitivity analyses were conducted using patients in the top 5% and top 20% of societal costs. Potential predictive factors included patient expectations, demographic factors (e.g. age, gender, nationality), socio‐economic factors (e.g. employment, education level) and health‐related factors (e.g. body mass index [BMI], general health, mental health). The final prediction models were obtained using backward selection. The model's prognostic accuracy (Hosmer–Lemeshow *X* ^2^, Nagelkerke's *R* ^2^) and discriminative ability (area under the receiver operating curve [AUC]) were assessed, and the models were internally validated using bootstrapping.

**Results:**

Poor physical health, high functional disability, low health‐related quality of life, high impact of pain experience, non‐Dutch nationality and decreasing pain were found to be predictive of high societal costs in all models, and were therefore considered robust. After internal validation, the models' fit was good, their explained variance was relatively low (≤14.1%) and their AUCs could be interpreted as moderate (≥0.71).

**Conclusion:**

Future studies should focus on understanding the mechanisms associated with the identified predictors for high societal costs in order to design effective cost reduction initiatives.

**Significance:**

Identifying low back pain patients who are at risk (risk stratification) of becoming high‐cost users and making appropriate initiatives could help in reducing high costs.

## 1 INTRODUCTION

In recent years, low back pain (LBP) has become the leading cause of years lived with disability in high‐, middle‐ and low‐income countries (Vos et al., 2017) A 54% increase in years lived with disability caused by LBP was reported worldwide between 1990 and 2015 (Hartvigsen, Hancock, & Kongsted, 2018) Next to the high disease burden of LBP, its economic burden is substantial (Tulder, Koes, & Bombardier, 2002) In 2007, for example, the societal cost of LBP in the Netherlands was estimated to be 3.5 billion euros, which accounted for approximately 0.6% of the Gross National Product (Lambeek et al., 2011). The estimated annual total cost of LBP in the United States is 100 billion dollars, (Dieleman et al., 2016) in Australia 9 billion Australian dollars, (Walker, Muller, & Grant, 2003) in Switzerland 6.6 billion euros (Wieser et al., 2011) and in the UK 12.3 billion British pounds (Maniadakis & Gray, 2000).

A systematic review by Hestbaek, Leboeuf‐Yde, and Manniche (2003) showed that in many cases LBP did not resolve on its own and that 62% of LBP patients keep experiencing pain after 12 months.(Hestbaek et al., 2003; Verkerk et al., 2013) Nonetheless, the majority of LBP patients do not seek treatment (Ferreira et al., 2010) and Engel, Von Korff, and Katon (1996) and Vlaeyen et al. (2018) reported that it is very likely that the majority of the total societal costs from LBP stem from a relatively small group of chronic LBP patients (Engel et al., 1996; Vlaeyen et al., 2018).

A proactive approach requires identifying high‐risk patients accurately before substantial avoidable costs have been incurred and health status has deteriorated. Exploring the mechanisms related to high‐cost users could potentially lead to ideas for initiatives or policy measures aimed at reducing costs. A report from The Commonwealth Fund (2012) maintains this view by placing emphasis on the need to address high‐cost health care users with chronic conditions if potentially significant gains are to be made (System TCFCoaHPH, 2012). Identifying factors predictive of high societal costs may provide opportunities to create appropriate initiatives aiming to prevent high‐cost outcomes as well as result in improvement of patient quality of life and a reduction in health care spending (Buchbinder et al., 2013; Chechulin, Nazerian, Rais, & Malikov, 2014).

To date, many studies have focused on investigating factors that predict whether acute LBP will become chronic. Various studies have identified a number of predictive factors for LBP chronicity, including high levels of psychological distress, low levels of physical activity, smoking, poor self‐rated health and dissatisfaction with employment (Klenerman, Slade, & Stanley, 1995; Linton & Halldén, 1998; Valat, Goupille, & Védere, 1997). However, in practice there is limited success in using this information to prevent or manage chronic LBP (Tulder et al., 2002; Waddell, 2006). Furthermore, whilst predictive factors have commonly been investigated in various other areas of LBP, such as identifying predictive factors for return to work, disability and future health care utilization, few studies have explored the possible factors that are predictive of high societal cost (Becker et al., 2010; Lancourt & Kettelhut, 1992; Pincus, Burton, Vogel, & Field, 2002; Skargren & Öberg, 1998). Therefore, the aim of this study was to identify predictive factors for high societal costs among chronic LBP patients in the Netherlands.

## 2 METHODS

### 2.1 Study population and design

A model was constructed to determine factors predicting high societal costs among chronic LBP patients. Data collected during the MinT (minimal invasive treatment) study in the Netherlands were used to develop the model. The MinT study consisted of three randomized controlled trials and an observational study. The aim of the MinT study was to assess the cost‐effectiveness of adding minimal interventional procedures to a standardized exercise program, compared with a standardized exercise program alone (Juch et al., 2017; Maas et al., 2012). Patients were eligible for the MinT study in general if they had chronic (>3 months) LBP, showed no improvement of symptoms after conservative treatment, were referred to a pain clinic and were able to complete Dutch questionnaires. Patients were included in the randomized controlled trials and observational study between 1 January 2013 and 1 July 2014 and between 1 January 2013 and 17 December 2015, respectively. In the present study, only data of the observational study were used. The observational study monitored patients who did not want to, or were not eligible to participate in the aforementioned randomized controlled trials or who received the intervention after recruitment for the randomized controlled trials was closed (between 1 July 2014 and 17 December 2015) (Maas et al., 2012). The exclusion criteria for participating in the randomized controlled trials included, amongst others, patients with a negative diagnostic test, patients with a body mass index (BMI) higher than 35, patients older than 70 years, patients with severe psychiatric or psychological problems, patients diagnosed with facet, disc, sacroiliac (SI) joint or combination pain but did not want to participate in the randomized controlled trials (Maas et al., 2012). The observational data will inform about the proportion of patients with a positive or negative diagnostic test for facet pain, disc pain, SI joint pain and a combination of these, and the clinical outcomes of patients with a negative diagnostic test. Patients diagnosed with facet, disc, SI joint or combination pain, by means of a diagnostic block, will be asked to take part of one of the four RCTs. The observational study will monitor patients who do not want to, or are not eligible to participate in the RCTs.

Ethical approval for the MinT study was obtained from the Medical Ethics Committee of the Erasmus Medical Centre in Rotterdam (registration number MEC‐2012–079). Local research governance was obtained from all participating pain clinics and all participants gave written informed consent (Maas et al., 2012).

### 2.2 Outcome measure

The outcome of the current study was having high societal costs (yes/no). Having high societal costs was defined as patients with costs in the top 10th percentile. Previous studies have defined high costs as patients in the top 20–25th percentile (Becker et al., 2010; Engel et al., 1996). A study in the United States studied health care expenditures from 1928 to 1996 found that the top 5% of high‐cost users accounted for more than half of health spending, while the top 10% accounted for about 70% of all health care spending (Berk & Monheit, 2001). For this study, the 10th percentile for societal costs was therefore assumed to be appropriate due to the large sample size.

Societal costs were measured using 3‐monthly retrospective cost questionnaires throughout the 1‐year study period (i.e. administered at 3‐, 6‐, 9‐ and 12‐month follow‐up; Goossens, Rutten‐van Mölken, Vlaeyen, & Linden, 2000). The self‐administered cost questionnaires included measures of health care utilization, informal care, unpaid productivity and absenteeism due to back pain. Health care utilization included primary care (e.g. general practitioner care, manual therapy, physical therapy, exercise therapy) and secondary care (e.g. diagnostic and therapeutic interventions, hospitalization). Data from the updated Dutch Manual of Costing were used to value costs of common health care services (Hakkaart‐van Roijen, Van der Linden, Bouwmans, Kanters, & Tan, 2015). For less common health care services, hospital accounting records and/or prices of professional organizations were used. Informal care and unpaid productivity were valued using the recommended Dutch shadow price of €14,32 per hour (Hakkaart‐van Roijen et al., 2015). Absenteeism from paid employment was measured using the Productivity and Disease Questionnaire (PRODISQ; Koopmanschap, 2005), and was valued in accordance with the friction cost approach using hourly productivity costs of males and females (Koopmanschap & Rutten, 1996). The friction cost approach assumes that production losses are confined to the period needed to replace a sick worker, which is currently assumed to be 12 weeks in the Netherlands (Hakkaart‐van Roijen et al., 2015). All costs were expressed in Euros 2017. An overview of the main cost categories, examples of common sub‐cost categories as well as their unit prices can be found in File S1.

### 2.3 Potential predictive factors

Potential predictive factors were based on previous literature (Becker et al., 2010; Chechulin et al., 2014; Engel et al., 1996; Klenerman et al., 1995; Lancourt & Kettelhut, 1992; Linton & Halldén, 1998; Pincus et al., 2002; Skargren & Öberg, 1998; Valat et al., 1997), and measured at baseline and included:

- Treatment credibility and patient expectancy for improvement after treatment (Credibility/Expectancy Questionnaire [CEQ] (Devilly & Borkovec, 2000); scores were transformed to 0—least credibility/expectancy to 100—more credibility/expectancy) to improve comparability of the odds ratios.
- Pain intensity (Numeric Pain Rating Scale [NPRS]; range 0—no pain to 100—worst pain imaginable; Childs, Piva, & Fritz, 2005). Scores were transformed to 0–100 to improve comparability of the odds ratios.
- Functional disability (Oswestry Disability Index [ODI]; range 0—no disability to 100—maximum disability; Davidson & Keating, 2005; Fairbank & Pynsent, 2000).
- Health‐related quality of life (EuroQol [EQ‐5D‐3L]; range 0—worst imaginable health state to 100—best imaginable health state, higher scores indicating better health; Rabin, 2001). The participants' EQ‐5D‐3L scores were converted into utility scores using the Dutch tariff (Lamers, Stalmeier, McDonnell, & Krabbe, 2005) and the scores were transformed to 0–100 to improve comparability of the odds ratios.
- General health—mental component score and physical component score (Rand‐36 [Rand‐36]; scores range 0—lowest general health to 100—highest general health) were transformed so that a higher score indicated better health status (Brazier et al., 1992; Hays & Morales, 2001; Vander Zee & Sanderman, 1996). The two dimensions of the Rand‐36 form, namely mental and physical health, were entered separately in the model.
- Impact of pain experience (Multidimensional Pain Inventory [MPI]; range 0—least/best to 100 most/worst). Scores were transformed to 0–100 to improve comparability of the odds ratios. For the purpose of this analysis, scores from the five sub‐scales of the first section of the MPI were used, that is, pain severity, interference with daily activities, life control, affective distress and support (Lousberg et al., 1999; McKillop & Nielson, 2011).
- Education level low/moderate/high. Low indicates no education, primary level education, lower vocational and lower secondary education; moderate indicates higher secondary education or undergraduate; high indicates tertiary education, university or postgraduate.
- Body mass index ([BMI], weight in kg/(height in metres)^2^).
- Employment (yes/no).
- Recurrent complaints (yes/no).
- Age in (years).
- Gender (male/female).
- Nationality (Dutch/non‐Dutch).
- Smoking (yes/no).
- Type of health care insurance (basic/additional).
- Region of residence (south/north/east/west).
- Married/living together (yes/no).
- Diagnosis (sacroiliac joint (SI)/facet/disc/combined/unclear). Diagnosis was based on medical history and clinical examination. Both followed a standard format and were performed by experienced clinicians. Depending on the suspected source of pain, clinical examination included provocation tests (compression test; distraction test; Flexion, Abduction, and External Rotation [FABER] test; Gaenslen test; thigh thrust test; Gillett test) and diagnostic anaesthetic blocks. For a more detailed description of the diagnostic procedures, we refer elsewhere (Juch et al., 2017; Maas et al., 2012).

### 2.4 Statistical analysis

The prediction model was constructed using multivariable logistic regression analysis (Harrell, Lee, & Mark, 1996; Steyerberg, 2010). Prior to constructing the model, missing data were handled using multiple imputation to avoid possible bias due to selective drop‐out of participants, which might influence the results when conducting a complete‐case analysis (Burton, Billingham, & Bryan, 2007). Imputations were performed by treatment group and per time point using predictive mean matching. Following this, tests were conducted to verify the linearity and additivity assumptions (Harrell et al., 1996).

Manual backward selection was used to obtain the final predictive factors with a *p* < .10. Variables with the highest *p*‐value were excluded from the model one by one and the analysis was rerun until only variables with a *p* < .10 constituted the model. A *p* < .10 was used to ensure that predictions are accurate, whilst preventing type‐1 errors caused by overfitting (Harrell et al., 1996). The overall performance and predictability of the model were tested using Nagelkerke's *R* ^2^ (Bewick, Cheek, & Ball, 2005; Greiner, Pfeiffer, & Smith, 2000; Steyerberg et al., 2010). Other performance measures included the area under the “receiver operating characteristics” (ROC) curve to measure the final model's discriminative value (area under the receiver operating curve [AUC]) (Bewick et al., 2005; Greiner et al., 2000; Steyerberg et al., 2010) as well as the Hosmer–Lemeshow goodness‐of‐fit to measure the calibration of the model (Bewick et al., 2005; Greiner et al., 2000; Steyerberg et al., 2010). To adjust for the fact that the model was developed and tested in the same population, which typically causes regression coefficients and performance measures to be overestimated (i.e. overfitting), bootstrapping was used to internally validate the model (Bewick et al., 2005; Greiner et al., 2000; Steyerberg et al., 2010). Multiple imputation and multivariate regression analyses were conducted using Stata (version 14SE, Stata Corp), and internal validation was performed using R (i386 version 3.1.2).

To test the robustness of the results, two sensitivity analyses were conducted; (a) using the top 20th percentile for high costs, and (b) using the top 5th percentile for high costs.

## 3 RESULTS

### 3.1 Participants

**Figure 1:**
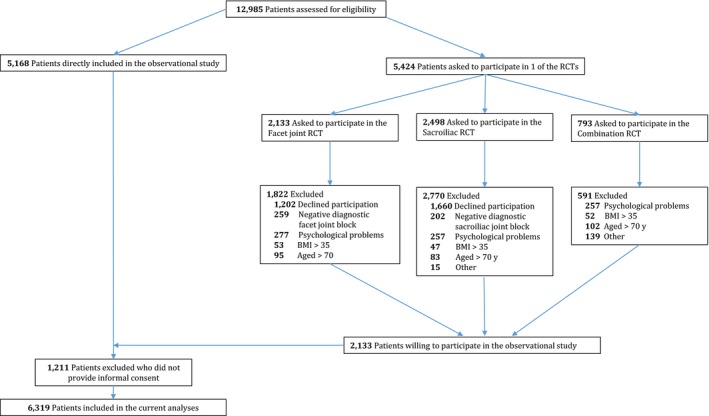
Inclusion and exclusion of participants

Data from 6,316 chronic LBP patients in the observational study group were analysed in the present study (Figure 1). Of them, the majority were female (66%), overweight (67%), Dutch (95%), had a low level of education (56%) and more than half were unemployed (59%; Table 1). Most of the predictive factors had about 17% of patients with missing data. The amount of missing values for all the variables entered in the model are reported in File S2. Costs at different cut‐off points were as follows: 10% (≥€11,922), 5% (≥€19,403) and 20% (≥€7,906). The average societal costs per patient were €5,522 and the median costs were €2,995.

**Table 1:** Patients' characteristics, all patients and according to societal costs (high vs. low)

Participant characteristic	All patients (*n* = 6,316)	High costs (*n* = 171)	Low costs (*n* = 6,145)
Age (years) [mean (*SD*)]	57.2 (13,4)	57.6 (12.0)	57.2 (13.5)
Gender (*n*, %)			
Female	4,142 (66)	128 (75)	4,014 (66)
Male	2,093 (34)	43 (25)	2,050 (34)
BMI (*n*, %)			
BMI < 18.5 (underweight)	37 (1)		37 (1)
BMI ≥ 18.5 < 25 (normal weight)	1,687 (32)	42 (26)	1,645 (32)
BMI ≤ 25 <30 (overweight)	2,060 (39)	62 (38)	1,998 (39)
BMI ≥ 30 (obese)	1,463 (28)	60 (37)	1,403 (28)
Smoking (*n*, %)			
Yes	1,413 (26)	42 (25)	1,371 (26)
No	3,920 (73)	125 (75)	3,795 (73)
Educational level (*n*, %)			
Low (no education, primary level education, lower vocational and lower secondary education)	2,925 (56)	100 (62)	2,825 (56)
Moderate (higher secondary education or undergraduate)	1,467 (28)	43 (27)	1,424 (28)
High (tertiary, university level, postgraduate)	830 (16)	19 (12)	811 (16)
Living together with a partner (*n*, %)			
Yes	4,663 (75)	135 (79)	4,528 (74)
No	1,593 (26)	36 (21)	1557 (26)
Nationality (*n*, %)			
Dutch	5,049 (95)	163 (98)	4,886 (95)
Non‐Dutch:	278(5.2)	4(2.8)	274(5.3)
Surinamese	21 (0.4)	0	21 (0.4)
Antillean/Aruban	22 (0.4)	0	22 (0.4)
Turkish	63 (1)	1 (1)	62 (1)
Moroccan	42 (1)	0	42 (1)
Other	130 (2.4)	3 (1.8)	127 (2.5)
Region in the Netherlands (*n*, %)			
South	2,029 (32)	59 (35)	1,970 (32)
North	1,165 (19)	30 (18)	1,135 (19)
East	1,280 (20)	43 (25)	1,237 (20)
West	1782 (28)	39 (23)	1743 (29)
Employment (*n*, %)			
Yes	1,687(42)	66 (39)	1,621 (42)
No	2,376 (59)	105 (61)	2,271 (58)
Recurrent low back pain (*n*, %)			
Yes	3,174 (63)	101 (62)	3,073 (63)
No	1876 (37)	61 (38)	1815 (37)
Diagnosis‐source of pain (*n*, %)			
1 = SI	1,864 (33)	57(36)	1,807 (33)
2 = Facet	2,269 (41)	54 (34)	2,215 (41)
3 = Disc	18 (0.3)	1 (0.63)	17 (0.3)
4 = Combined	1,391 (25)	44 (28)	1,347 (25)
5 = Unclear	66 (1)	3 (2)	63 (1)
Patients expectations			
Credibility [mean (*SD*)] range 0–100	77.1 (17.5)	77 (19.1)	77.1 (17.3)
Expectancy [mean (*SD*)] range 0–100	57.8 (17.3)	57.2 (17)	58 (17)
Rand − 36			
Mental [mean (*SD*)] range 0–100	22.6 (5)	21.6 (5)	22.6 (5)
Physical [mean (*SD*)] range 0–100	18.5 (4)	16.0 (4)	18.6 (4)
Health‐related quality of life(utility) [mean (*SD*)] range 0–100	48 (29)	31 (28)	48 (29)
MPI [mean (*SD*)] range per subscale 0–100			
Pain severity	22.6 (5.7)	25.4 (4.4)	22.5 (5.7)
Interference with daily activities	5.8 (1.9)	6.9 (1.6)	5.8 (1.9)
Life control	21.2 (6.3)	20.2 (7)	21.2 (6.2)
Affective distress	15.4 (4.6)	16.5 (4.9)	15.3 (4.6)
Support	28.6 (7.6)	30.4 (6.2)	28.4 (7.6)
Type of health care insurance (*n*, %)			
Basic insurance	633 (12)	14 (8)	619 (12)
Comprehensive (basic + additional cover)	4,630 (86)	153 (92)	4,477 (86)
I don't know	55 (1)	0	55 (1)
ODI functional disability [mean (*SD*)] range 0–100	11.1 (9)	17.1 (10)	11.1 (9)
Pain intensity [mean (*SD*)] range 0–100	73 (16)	77 (14)	73 (16)

Note

Percentages have been rounded off hence values a bit less than 100% and a bit more that 100%. Scores for MPI, Rand 36, patient expectations, health‐related quality of life were transformed to a range of 0–100 to enable comparability with the odds ratio. Diagnosis was based on patient history and physical examination.

Abbreviations: MPI, multidimensional pain inventory; ODI, oswestry disability index.

### 3.2 Development, performance and internal validity of the top 10% prediction model

Females, non‐Dutch nationals, combined diagnosis (LBP caused by both facet joints and intervertebral disc), poor physical health, high functional disability, low health‐related quality of life, decreasing age, high impact of pain experience and decreasing pain intensity were found to increase the odds of having high societal costs (Table 2). The Hosmer–Lemeshow statistic was not significant (*X* ^2^ = 7, *p* = .55), indicating that the model's overall fit was good. The model explained 14.3% (Nagelkerke's *R* ^2^) of the variation in the outcome (i.e. high societal costs) and the model's AUC was 0.74 (95% CI 0.67–0.72). After internal validation, the model's explained variance was 13.2% and the AUC was 0.73. The calibration slope was 0.97, indicating relatively little optimism or overfitting of the regression coefficients.

**Table 2:** Multivariate model using the top 10th percentile of societal costs as an outcome

	Coefficient (regression)^a^	SE (of regression coefficient)	*p*‐value	95% CI		
				Lower bound	Odds ratio	Upper bound
Diagnosis (ref: sacroiliac joint)						
Facet	0.097	0.139	0.487	0.836	1.102	1.452
Disc	0.109	0.983	0.912	0.161	1.115	7.744
Combined	0.263	0.142	0.066	0.982	1.301	1.725
Unclear	0.731	0.442	0.100	0.868	2.077	4.972
Physical health (Rand − 36); range 0–100	−0.069	0.021	0.002	0.895	0.933	0.973
Functional disability (ODI); range 0–100	0.035	0.008	0.000	1.019	1.036	1.053
Health‐related quality of life (EQ−5D−3L); range 0–100	−0.006	0.029	0.052	0.989	0.994	1.000
Impact of pain experience (MPI interference) range 0 − 100	0.188	0.051	0.000	1.092	1.207	1.336
Nationality (ref: non‐Dutch)	−0.818	0.215	0.000	0.286	0.441	0.680
Pain intensity (NPRS) range 0–100	−0.011	0.004	0.010	0.981	0.989	0.997
Age (years)	−0.009	0.004	0.031	0.982	0.991	0.999
Gender (ref: female)	−0.214	0.111	0.055	0.649	0.807	1.004
Constant	−0.392	0.762	0.608	0.148	0.676	3.080

Abbreviation: CI, confidence interval; MPI, multidimensional pain inventory; NPRS, numeric pain rating scale; ODI, oswestry disability index; SE, standard error.

a Coefficient multivariable logistic regression

### 3.3 Sensitivity analysis

Using an outcome consisting of patients in the top 20th percentile of societal costs, combined diagnosis, poor physical health, high functional disability, low health‐related quality of life, high impact of pain experience, non‐Dutch nationality, decreasing pain intensity and being female were predictive factors of having high societal costs (Table 3). The Hosmer–Lemeshow statistic was not significant (*X* ^2^= 8.5, *p* = .47), Nagelkerke's *R* ^2^ was 0.146 and the model's AUC was 0.72. After internal validation, the model's explained variance reduced to 14.1% and the AUC to 0.71. The calibration slope was 0.98.

**Table 3:** Multivariate model using the top 20th percentile of societal costs as an outcome

	Coefficient (regression)^a^	SE (regression coefficient)	*p*‐value	95% CI		
				Lower bound	Odds ratio	Upper bound
Diagnosis (ref: sacroiliac joint)						
Facet	0.053	0.113	0.640	0.841	1.054	1.322
Disc	0.355	0.722	0.624	0.342	1.426	5.954
Combined	0.280	0.110	0.013	1.063	1.323	1.649
Unclear	0.297	0.378	0.433	0.638	1.346	2.843
Physical health (Rand−36); range 0–100	−0.056	0.014	0.000	0.919	0.946	0.973
Functional disability (ODI); range 0–100	0.028	0.007	0.000	1.015	1.028	1.043
Health‐related quality of life (EQ−5D−3L); range 0–100	−0.005	0.002	0.006	0.991	0.995	0.998
Impact of pain experience (MPI interference) range 0–100	0.176	0.036	0.000	1.110	1.192	1.280
Nationality (ref: non‐Dutch)	−0.948	0.226	0.000	0.244	0.388	0.616
Pain intensity (NPRS); range 0–100	−0.010	0.003	0.002	0.984	0.990	0.996
Gender (ref: female)	−0.239	0.089	0.008	0.660	0.787	0.939
Constant	−0.027	0.559	0.962	0.318	0.973	2.983

Abbreviation: CI, confidence interval; MPI, multidimensional pain inventory; NPRS, numeric pain rating scale; ODI, oswestry disability index; SE, standard error.

a Coefficient multivariable logistic regression.

Using an outcome consisting of patients in the top 5th percentile of societal costs, high‐level education, poor physical health, high functional disability, low health‐related quality of life, high impact of pain experience, non‐Dutch nationality and decreasing pain intensity were predictive factors of having high societal costs (Table 4). The Hosmer–Lemeshow statistic was not significant (*X* ^2^ = 7.2, *p* = .59), indicating that the model's overall fit was good. The model explained 14.1% (Nagelkerke's *R* ^2^) of the variation in the outcome (high costs) and the model's AUC was 0.76. After internal validation, the model's explained variance reduced to 13.2% and the AUC to 0.76. The calibration slope was 0.97.

**Table 4:** Multivariate model using the top 5th percentile of societal costs as an outcome

	Coefficient (regression)^a^	SE (regression coefficient)	*p*‐value	95% CI		
				Lower bound	Odds ratio	Upper bound
Education (ref: low)						
Medium	0.099	0.201	.626	0.738	1.104	1.650
High	0.396	0.227	.086	0.940	1.486	2.336
Physical health (Rand−36); range 0–100	−0.078	0.026	.004	0.878	0.925	0.974
Functional disability (ODI); range 0–100	0.041	0.011	0000	1.019	1.042	1.065
Health‐related quality of life (EQ−5D−3L); range 0–100	−0.008	0.003	.028	0.986	0.992	0.999
Impact of pain experience (MPI interference) range 0–100	0.183	0.063	.004	1.061	1.201	1.359
Nationality (ref: non‐Dutch)	−0.855	0.248	.001	0.259	0.425	0.697
Pain intensity (NPRS); range 0–100	−0.013	0.006	.023	0.975	0.987	0.998
Constant	−1.413	0.904	.121	0.040	0.243	1.463

Abbreviation: CI, confidence interval; MPI, multidimensional pain inventory; NPRS, numeric pain rating scale; ODI, oswestry disability index; SE, standard error.

a Coefficient multivariable logistic regression

Table 5 provides an overview of robust predictors of high societal costs in all three models

**Table 5:** Robust predictors of high societal costs in all three models

	Top 10th percentile	Top 20th percentile	Top 5th percentile
	Odds ratio (95% CI)	Odds ratio (95% CI)	Odds ratio (95% CI)
Physical health (Rand − 36); range 0–100	0.933 (0.895–0.973)	0.946 (0.919–0.973)	0.926 (0.878–0.976)
Functional disability (ODI); range 0–100	1.036 (1.019–1.053)	1.028 (1.015–1.043)	1.041 (1.018–1.063)
Health‐related quality of life (EQ−5D−3L); range 0–100	0.994 (0.989–1.000)	0.995 (0.991–0.998)	0.992 (0.985–0.999)
Impact of pain experience (MPI interference) range 0–100	1.017 (1.008–1.027)	1.016 (1.010–1.016)	1.017 (1.005–1.028)
Nationality (ref: non‐Dutch)	0.441 (0.286–0.680)	0.388 (0.244–0.616)	0.424 (0.258–0.698)
Pain intensity (NPRS); range 0–100	0.989 (0.981–0.997)	0.990 (0.984–0.996)	0.987 (0.976–0.998)

Abbreviation: CI, confidence interval; MPI, multidimensional pain inventory; NPRS, numeric pain rating scale; ODI, oswestry disability index.

## 4 DISCUSSION

### 4.1 Main findings

High impact of pain experience (MPI interference), being female, non‐Dutch national, combined diagnosis (LBP caused by both facet joints and intervertebral disc), poor physical health, high functional disability, low health‐related quality of life, younger age and decreasing pain intensity were found to increase the odds of having high societal costs. The model's overall fit was good and its explained variance was relatively low (Bewick et al., 2005; Greiner et al., 2000; Steyerberg et al., 2010; that is, only 14.3% of the variance in high societal costs was explained by the identified predictive factors). The AUC was 0.73 and can be interpreted as moderate (Greiner et al., 2000). Internal validation had little effect on the model's performance, illustrating minimal chance of overfitting of the regression coefficients (Steyerberg et al., 2010).

At a 5% cut‐off point in our sensitivity analysis, high education level became a predictor and gender and age were no longer predictors. There were no additional predictive factors when a cut‐off point of 20% was used, instead age was no longer a predictor. The performance of the sensitivity analyses models was equal to that of the main analysis. Poor physical health, high functional disability, low health‐related quality of life, high impact of pain experience, non‐Dutch nationality and decreasing pain were found to be predictive of having high societal costs in all models, suggesting that they constitute the most robust predictors of high societal costs.

### 4.2 Comparison with literature

Few studies have focused on investigating predictive factors for high societal costs among chronic LBP patients. A study by Engel et al. (1996) reported increasing chronic pain grade and pain persistence as strong predictors of high costs and high back pain costs, followed by disc disorder/sciatica diagnosis and increasing depressive symptoms. Diagnosis as a predictor of high costs is in line with the results of the present study as well as those of previous ones (Becker et al., 2010; Wenig, Schmidt, Kohlmann, & Schweikert, 2009). In contrast to the present study, they found mental health and high pain scores to be predictors for high costs. Mental health was also a predictor of high societal costs in the studies of Becker et al. (2010) and Ritzwoller, Crounse, Shetterly, and Rublee (2006). This discrepancy could be due to different cut‐off points for high costs (>20% in the previous studies vs. 10% in the present study). The definition of mental health (i.e. depression vs. general mental health) varied among the studies, Becker et al. (2010) focused on depression, whereas Ritzwoller et al. (2006) included anxiety, depression and psychosis. Differences in measuring mental health were noted, 1‐item question (present study) versus a risk adjustment system used to identify comorbidities (Ritzwoller et al., 2006) versus CES‐D ranging from 0 to 60 (Becker et al., 2010). Depression was associated with high health care costs in the study of Becker et al. (2010) and a possible explanation was that physicians initiate costly health care when confronted with mood disorders (Becker et al., 2010). Ritzwoller et al. (2006) reported an association of depression and psychopathy with increased LBP episodes and high costs. Comorbidities have been associated with longer duration of LBP and work disabilities (Nordin et al., 2002).

Although previous studies have reported an increase in LBP intensity to be a predictor of high costs (Becker et al., 2010; Wenig et al., 2009), the present study reported decreasing pain intensity as a predictor of high costs. A possible explanation for this discrepancy is that only chronic LBP was included in the present study versus general LBP (acute and chronic; Becker et al., 2010; Ekman, Jönhagen, Hunsche, & Jönsson, 2005; Engel et al., 1996; Ritzwoller et al., 2006; Wenig et al., 2009) and that the studies took place in different health care settings, that is, primary (Becker et al., 2010; Ekman et al., 2005; Engel et al., 1996; Ritzwoller et al., 2006) versus secondary (present study). Fink‐Miller, Long, and Gross (2014) reported that chronic LBP patients in primary care reported more severe pain compared to chronic LBP patients in tertiary care and suggest shorter duration of complaints and shopping for opioids by chronic LBP patients in primary care as possible explanations (Fink‐Miller et al., 2014). Also, patients presenting in secondary and/or tertiary care may have exhausted conservative therapies, hence could have already made high costs.

Contrary to the findings of Wenig et al. (2009), being female was a predictor of high costs in the present study and in previous studies (Ekman et al., 2005). Wenig et al. (2009) reported that women had a higher probability to cause high costs and utilized health care more quickly than men and when men used health care for LBP it resulted in higher costs on average. The present study had almost double the amount of women compared to men, whereas there was a small difference in the amount of men and women in the study of Wenig et al. (2009).

Another important difference between the present study and the previous ones is the applied perspective. In the present study, a societal perspective was applied, including health care, absenteeism, informal care and unpaid productivity costs, whereas Engel et al. (1996) and Ritzwoller et al. (2006) only included health care costs. Becker et al. (2010) evaluated costs from a societal perspective but did not include informal care costs, Wenig et al. (2009) also applied a societal approach that included health care and lost productivity costs, but did not include informal care costs.

Also important to note is the higher Nagelkerke's *R* ^2^ for the model by Becker et al. (i.e. 0.28) compared to that of the present study (i.e. 0.14). Information regarding the fit of the model (Nagelkerke's *R* ^2^, AUC) is missing from some previous studies (Engel et al., 1996; Wenig et al., 2009). In the present study, the explained variance was probably lower than that of other studies because we applied the broadest perspective, that is, the societal one. The relatively low explained variance may also be interpreted as the variables entered into our model are less suitable at predicting high costs (Ekman et al., 2005), important predictors are missing or chronic LBP patients who are having high costs are a heterogeneous population. Demographic, social and clinical factors included in this model, as in other prediction studies, are typically measured in LBP studies.

Other predictive factors of high costs include diabetes, rheumatoid arthritis, back pain persistence (Engel et al., 1996), fear of avoidance beliefs (Becker et al., 2010), low education and unemployment (Wenig et al., 2009). In contrast to our findings, both low education level and unemployment were not predictors of high costs in our sensitivity analysis, but high education level was. A possible explanation for this is that, 86% of the patients included in this study had comprehensive health care insurance. Highly educated persons are likely to afford more expensive and comprehensive insurance packages offering more options for health care and visits to alternative medicine and therapies. This finding has important implications for the understanding of the relation between socio‐economic status and high‐cost users in chronic LBP. In addition, for interventions and policies aimed at highly educated high‐cost users in LBP.

In the present study, the average societal costs per patient were €5,522, whereas Dutmer et al (2019) reported around €9,000 in societal costs per patient (Dutmer et al., 2019). This difference could have resulted from the absence of presenteeism costs in the present study, whereas Dutmer et al (2019) did include this cost category in their societal cost estimation. As a consequence, some productivity costs may have been missed. In addition, only patients from a secondary setting were included in the present study, whereas Dutmer et al (2019) included patients from both secondary and tertiary settings. Tertiary settings are generally more costly compared to secondary settings. Moreover, Dutmer et al (2019) reported higher levels of disability than were reported in the present study, while high levels of disability are typically associated with high costs in LBP (Hartvigsen et al., 2001; Lambeek et al., 2011).

### 4.3 Strength and limitations

Strengths of the present study include that it was one of the very few studies to identify predictive factors for high costs in patients with chronic LBP and that the societal perspective was applied. The large cohort of observed patients with chronic LBP (*n* = 6,316) greatly increases the power of this study and improves sensitivity to weak predictive factors. Imputation methods were used to deal with missing data thereby avoiding complete‐case analysis which would have significantly reduced the power of these findings and potentially introduced information bias due to selective drop‐out of participants. Multiple imputation is the preferred statistical method for dealing with missing data, particularly when costs are involved (Burton et al., 2007). Furthermore, internally validating the model by bootstrapping with 250 replications improved the generalizability and robustness of these findings (Bewick et al., 2005; Steyerberg et al., 2013).

Some limitations are notable as well. Although mainly valid and reliable questionnaires were used, the predictive factors were measured using self‐reported questionnaires and this might have caused recall and or social desirability bias. Second, presenteeism costs were not included in our analyses, whereas presenteeism has previously been found to be a very important cost driver and is increasingly being recognized as an important problem in the occupational setting (Tsuboi, Murata, Naruse, & Ono, 2019). Hence, further productivity losses could have been missed. Future studies should therefore include presenteeism costs. Third, there is no consensus regarding the most ideal cut‐off point for defining high costs. Although in this study different cut‐off points, that is, 10% (≥€11,922), 5% (≥€19,403) and 20% (≥€7,906), were used to assess the robustness of the model, a consensus should be reached on the definition of high costs. This will enable the results to be more comparable and also determine the most suitable moment for initiatives aiming to reduce these costs to be applied. Fourth, in spite of the relatively large sample size of the current study (*n* = 6,316), there were some predictive factors for which there were very few participants. For example, there were only four (2.8%) non‐Dutch nationals in the high‐cost group in the main analysis, and it is unknown whether these four participants are representative of all non‐Dutch LBP patients. As a consequence, even though non‐Dutch nationality was identified as a predictor in all of the models, further research is needed to establish whether non‐Dutch nationality is indeed a very strong predictor of having high societal costs among LBP patients. Fifth, the secondary care setting of this study may to some extent limit the generalizability of its findings to other types of LBP patients and/or other settings. Amongst others, the relatively high unemployment rate of 59% may have resulted in an underestimation of the productivity costs, whereas secondary care is generally more expensive than primary care and health care costs may thus have been overestimated (Lambeek et al., 2011). As a consequence, the total societal cost estimates are likely to be specific to the secondary care setting. Furthermore, the disability rate in this study is rather low in comparison to other studies conducted in secondary settings (Dutmer et al., 2019), therefore caution should be exercised when applying these results to other populations. Sixth, apart from high BMI‐related diseases no other comorbidities have been included in the study. Overweight and obesity are well represented in the present study because these were exclusion criteria for the RCTs in the Mint study.

### 4.4 Implications for research and practice

The lack of professional consensus regarding a cut‐off point for high costs is probably due to limited studies in this field. Having a consensus regarding a cut‐off point can enable comparisons to be made and it is essential in policy and decision making. Identifying those patients who are at risk (risk stratification) of becoming high‐cost users and making appropriate initiatives could help in reducing high costs. For example, non‐Dutch nationality might be associated with a more limited mastery of the language. Maybe the information provided to non‐Dutch patients should be adapted. Functional disability and poor physical health are predictors of high societal costs, therapies targeting limitations in activities could play a role in reducing societal costs. There is evidence from randomized controlled trials that stratified care models limit long‐term disability arising from LBP (Linton, Nicholas, & Shaw, 2018). These considerations have important implications for how the link between socio‐economic status and high‐cost use is understood and for policies and programs targeting high‐cost use.

## 5 CONCLUSION

The present study identifies patients at risk of becoming high‐cost users and future studies should focus on understanding the mechanisms associated with the identified predictors for high‐cost users in order to be able to design and tailor effective cost reduction initiatives.

## AUTHOR CONTRIBUTIONS

EM wrote the initial version of the manuscript. EM, ML, JvD and ET were involved in the data analysis process. All authors discussed the results and commented on the manuscript. FH, MvT and RO received funding for the study.
