# Supplementary material for: Predictive factors of high societal costs among chronic low back pain patients
Source: Eur J Pain. 2019 Oct 10;24(2):325–37. doi: 10.1002/ejp.1488 (PMC7003839; doi:10.1002/ejp.1488)
Supplement: Supplementary file 1 [file EJP-24-325-s001.docx]

***S1****. Main cost categories, examples of common sub-cost categories, and unit prices.*

| **Main cost categories** | **Costs included** | **Common sub-cost categories** | **Unit prices**  **(Euros 2017)** |
| --- | --- | --- | --- |
| Healthcare costs | Primary healthcare costs related to the participants chronic low back pain | General practitioner | €33.76/visit |
|  |  | Physiotherapy | €33.76/visit |
|  |  | Social worker | €59.01/visit |
|  |  | Psychologist | €94.45/visit |
|  |  | Manual therapist | €38.79/visit |
|  |  | Chiropractor | €50.37/visit |
|  |  | Ergotherapy | €33.76/visit |
|  |  | Haptotherapy | €77.69/visit |
|  |  | Podotherapy | €60.93/visit |
|  |  | Accupuncture | €50.37/visit |
|  |  | Ceasar therapy | €34.79/visit |
|  |  | Magnetizer | €28.65/visit |
|  |  | Massage | €42.65/visit |
|  | Secondary health care costs related to the participants chronic low back pain | Hospital admission | €502.26/day |
|  |  | Day treatment in hospital | €226.63/day |
|  |  | Policlinic visits | €81.85/visit |
|  |  | Intensive care treatment | €2399.20/day |
|  |  | Community/home care | €26.45/hour |
|  |  | Radiofrequency denervation | Facet joint: €927/treatment  Sacroiliac joint: €815/treatment |
|  |  | Diagnostic block | €253/treatment |
| Informal care costs | Costs related to all hours of care provided by family, friends, and other volunteers due to the participants’ chronic low back pain |  | €14.32/hour |
| Unpaid productivity costs | Costs related to all hours of volunteer work, domestic and educational activities that participants were not able to perform due to their chronic low back pain |  | €14.32/hour |
| Absenteeism costs | Costs related to all hours absence from work due to the participants’ chronic low back pain |  | Male: €38.78/hour  Female: €32.33/hour |
