# Supplementary material for: Predictive factors of high societal costs among chronic low back pain patients
Source: Eur J Pain. 2019 Oct 10;24(2):325–37. doi: 10.1002/ejp.1488 (PMC7003839; doi:10.1002/ejp.1488)
Supplement: Supplementary file 2 [file EJP-24-325-s002.docx]

**S2**. Potential predictive variables and the amount of missing variables.

| Variables | Number of missing values | Number of non-missing values | Percentage of missing values |
| --- | --- | --- | --- |
| Education | 1094 | 5222 | 17 |
| Diagnosis | 708 | 5608 | 11 |
| Patients Expectations | 1062 | 5254 | 17 |
| SF-36: Mental health | 1068 | 5248 | 17 |
| SF-36 :Physical health | 1063 | 5253 | 17 |
| BMI | 1069 | 5247 | 17 |
| Health related quality of life (utility) | 1055 | 5261 | 17 |
| MPI: Pain severity | 1069 | 5247 | 17 |
| MPI: Interference with daily activities | 1075 | 5241 | 17 |
| MPI: Life control | 1075 | 5241 | 17 |
| MPI: Affective distress | 1075 | 5241 | 17 |
| MPI: Support | 1071 | 5245 | 17 |
| NPRS: Pain | 1054 | 5262 | 17 |
| Employment | 2253 | 4063 | 36 |
| Chronic complaints | 1070 | 5246 | 17 |
| Recurrent complaints | 1266 | 5050 | 20 |
| Age | 982 | 5334 | 16 |
| Sex | 81 | 6235 | 16 |
| Dutch nationality | 989 | 5327 | 16 |
| Smoking | 955 | 5361 | 16 |
| Type of health care insurance | 955 | 5361 | 16 |
| Region in Netherlands | 60 | 6256 | 1 |
| Marital status | 60 | 6256 | 1 |
| ODI: Functional disability | 1059 | 5257 | 20 |
